# Results from a retrospective case finding and re-engagement exercise for people previously diagnosed with hepatitis C virus to increase uptake of directly acting antiviral treatment

**DOI:** 10.1186/s12889-024-19919-3

**Published:** 2024-09-06

**Authors:** David Etoori, Ruth Simmons, Monica Desai, Graham R. Foster, Avelie Stuart, Caroline Sabin, Sema Mandal, William Rosenberg

**Affiliations:** 1 Centre for Clinical Research, Epidemiology, Modelling and Evaluation (CREME), Institute for Global Health, University College London, Royal Free Campus, Rowland Hill Street, London, NW3 2PF UK; 2 National Institute for Health and Care Research (NIHR) Health Protection Research Unit (HPRU) in Blood-borne and Sexually Transmitted Infections at UCL in partnership with the UK Health Security Agency (UKHSA), Royal Free Campus, Rowland Hill Street, London, NW3 2PF UK; 3 Sexually Transmitted Infections and HIV Division, Blood Safety, Health Security Agency, 61 Colindale Avenue, NW9 5EQ Hepatitis, London, UK; 4 Barts Liver Centre, The Blizard Institute, QMUL, London, UK

**Keywords:** HCV, Retrospective, Case-finding, Evaluation

## Abstract

**Background:**

Direct acting antivirals (DAAs) for the Hepatitis C virus (HCV) have shifted the World Health Organisation global strategic focus to the elimination of HCV by 2030. In England, the UK Health Security Agency (UKHSA) led a national ‘patient re-engagement exercise’, using routine surveillance data, which was delivered through the HCV Operational Delivery Networks (ODNs) with support from National Health Service England (NHSE), to help find and support people with a positive HCV PCR test result to access treatment. We report a quantitative evaluation of outcomes of this exercise.

**Methods:**

Individuals with a recorded positive HCV antibody or PCR result between 1996 and 2017 were identified using UKHSA’s records of HCV laboratory diagnosis. Linkage with established health-care datasets helped to enhance patient identification and minimise attempts to contact deceased or previously treated individuals. From September to November 2018 each ODN was provided with a local list of diagnosed individuals. ODNs were asked to perform further data quality checks through local systems and then write to each individual’s GP to inform them that the individual would be contacted by the ODN to offer confirmatory HCV PCR testing, assessment and treatment unless the GP advised otherwise. Outcomes of interest were receipt of treatment, a negative PCR result, and death. Data were collected in 2022.

**Results:**

Of 176,555 individuals with a positive HCV laboratory report, 55,329 individuals were included in the exercise following linkage to healthcare datasets and data reconciliation. Participants in the study had a median age of 51 years (IQR: 43, 59), 36,779 (66.5%) were males, 47,668 (86.2%) were diagnosed before 2016 and 11,148 (20.2%) lived in London. Of the study population, 7,442 (13.4%) had evidence of treatment after the re-engagement exercise commenced, 6,435 (11.6%) were reported as PCR negative (96% had no previous treatment records), 4,195 (7.6%) had prescription data indicating treatment before the exercise commenced or were reported to have been treated previously by their ODN, and 2,990 (5.4%) had died. The status of 32,802 (59.3%) people remains unknown.

**Conclusions:**

A substantial number of those included had treatment recorded after the exercise commenced, however, many more remain unengaged. Evaluation of the exercise highlighted areas that could be streamlined to improve future exercises.

**Supplementary Information:**

The online version contains supplementary material available at 10.1186/s12889-024-19919-3.

## Background

The introduction of direct acting antiviral (DAA) treatments for the Hepatitis C virus (HCV), which are known to cure HCV in the majority of those treated [1–3], shifted the World Health Organisation (WHO) global strategy to the elimination of HCV (curing 80% of those diagnosed) as a public health threat by 2030 [4]. The UK government has committed to this strategy, with the National Health Service England (NHSE) having an ambition to eliminate HCV ahead of the 2030 goal [5–7]. Part of this commitment involves efforts to re-engage people previously diagnosed but no longer actively accessing services, aligning with targets to reduce both incidence of and mortality from viral hepatitis [8]. 

HCV infection in England is primarily driven by injecting drug use, the reported risk factor for 77% of infections, with a third of people living with chronic HCV currently injecting drugs [9]. Engagement in services for people who inject drugs (PWID) was historically low and characterised by mistrust and discrimination [10]. 

There have been significant efforts to (re)test, (re)diagnose and (re)treat those living with chronic HCV since DAAs became widely available. In England, between 2016 and 2021, 73% of people with a positive HCV RNA result had a record indicating DAA treatment initiation and 47% had a record indicating sustained virologic response (SVR) (HCV not detected in the blood 12 weeks after completing treatment) [6]. As a result, and through concerted efforts by partners across community, government and non-government groups, modelling estimates show a 45% decrease in HCV prevalence in England since 2015 [6]. Mortality and morbidity associated with end stage liver disease (ESLD) and hepatocellular carcinoma (HCC) have declined in England, surpassing the WHO’s target for reduction in mortality (10% reduction between 2015 and 2020, and 65% by 2030) [9]. Successful treatment has also led to significant improvements in quality of life [11]. 

However, England had a large population of people known to be living with chronic HCV before the advent of DAAs [9]. The frequently asymptomatic nature of HCV infection meant that many individuals may not have been aware that they had previously acquired HCV. Prior to DAA availability, the limited treatment options had poor side effect profiles and relatively poor outcomes [12, 13]. As a result, many people living with HCV never accessed treatment services or, if they did, were unable to complete treatment and/or subsequently disengaged with services [14]. To support people previously diagnosed with HCV to be treated for their infection with DAAs, the UK Health Security Agency (UKHSA), in partnership with NHSE, launched a national ‘HCV patient re-engagement exercise’ to help find and support engagement in care for these individuals by collaborating with the Operational Delivery Networks (ODNs). ODNs are formal NHSE structures in which providers, commissioners and patients work together to optimise healthcare including routine care and treatment of people with HCV infection. ODNs mainly focus on coordinating patient pathways between providers over a wide area to ensure patients’ access to specialist resources and expertise [15]. This re-engagement exercise was launched in collaboration with peer support and patient advocacy groups who co-produced patient-facing resources, GP letters and outreach services [15]. 

In this paper we (a) describe the implementation of the HCV re-engagement exercise, and (b) report on the quantitative process and outcome evaluation of using laboratory surveillance data as a prompt for re-engagement into HCV care and increasing treatment uptake.

## Methods

### Laboratory surveillance data

Routine laboratory reports of HCV diagnosis (defined as the detection of HCV antibody (anti-HCV) and/or HCV RNA) to UKHSA by diagnostic laboratories were used to identify individuals diagnosed with HCV between 1996 and 2017 (*n* = 176,555).

Laboratory HCV reports have been submitted to UKHSA and predecessor organisations from NHS laboratories through surveillance forms or electronically since 1990, with better coverage and more streamlined reporting systems since 1996. Reporting completeness further improved when laboratory notification of a diagnosis of viral hepatitis became mandatory in 2010. It is not possible to consistently distinguish those with active infection (based on a positive HCV RNA result) from those with cleared infection as the system collects mainly anti-HCV results, and so laboratory ‘confirmed’ cases are a mixture of those with current and cleared infections. Laboratory reports include basic demographics.

### Data processing and linkage to generate patient lists

**Fig. 1:**
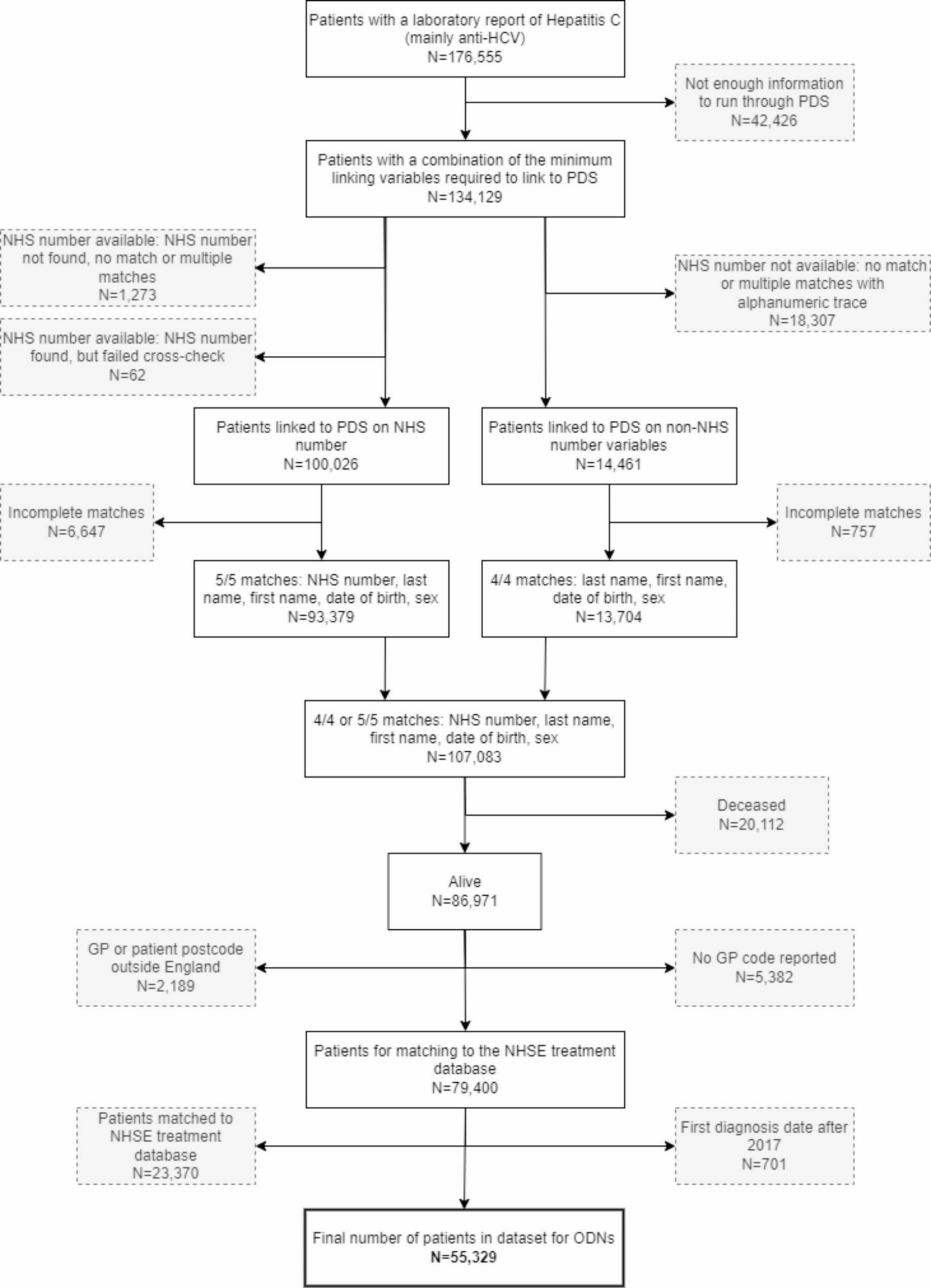
Data flow and matching steps to generate the lists for ODNs. * Linking variables: NHS number, first name, last name, date of birth, and sex

A number of steps were taken to clean, reconcile and link data to generate patient lists. (Fig. 1) Of the 176,555 individuals with an HCV diagnosis recorded since 1996, 42,426 were excluded as minimal identifiable data was not available (i.e., name, date of birth, sex, NHS number). As laboratory data were submitted to UKHSA for surveillance purposes rather than for direct patient care, the reporting and completeness was variable. Data completeness was 80.2% for last name, 80.5% for first name, 98.2% for date of birth, 98.0% for sex, and 63.8% for NHS number.

We performed three linkage steps. First, individuals with names, date of birth, and/or NHS number (134,129) were linked to the NHS spine (Personal Demographics Service (PDS) – the national master database of all NHS patients in England) to cross-check identifiable information and to identify their current registered GP. Anyone in England can register with a GP to access NHS services [16]. However, this is not compulsory. In total, 100,026 individual records were linked to the spine via NHS number, and 14,461 were linked through alphanumeric matches. Of these, 7,404 were excluded due to a non-perfect link on name, date of birth or sex between the information reported on the spine and that reported with the diagnosis record.

Second, through linking to registered deaths provided by the Office of National Statistics (ONS) and/or a died flag in the NHS spine, 20,112 of the 107,083 individuals believed to have died were excluded, with a further 7,571 excluded who were not registered with a GP or who registered with a GP outside England.

Finally, the remaining individuals (79,400) were linked to the HCV patient registry and treatment outcome database (NHSE registry), which stores records of all individuals referred to ODNs for DAAs. A further 23,370 individuals already known to the ODNs were excluded as a result, as were those first diagnosed after 2017 (*n* = 701), leaving details of 55,329 individuals to be distributed to the ODNs. Individuals were assigned to an ODN using their current residential postcode or registered GP postcode retrieved through linking to the NHS Spine.

Additional flags were added to the patient lists if an individual was also found on other surveillance and healthcare datasets e.g. sentinel surveillance of blood-borne virus testing (SSBBV), Hospital Episode Statistics (HES), the NHS Blood and Transplant (NHSBT) registry, to add assurance of further evidence of HCV testing and care, to validate personal confidential data (name, date of birth, sex, NHS number) and to avoid sending letters to individuals not requiring any intervention (e.g., those who had already had a liver transplant).

All data processing and linkage was done using Microsoft SQL Server.

### Implementation

Between September - November 2018, UKHSA, through a secure electronic file transfer platform, provided each ODN with a list of eligible individual residents in their ODN area. UKHSA published guidance to GPs and ODNs, patient and GP leaflets, and template letters, co-developed with the Hepatitis C Trust and NHSE, to support the re-engagement exercise [15]. 

ODNs undertook further quality checks of the data with their local IT systems (e.g., laboratory, patient administrative, and treatment databases) to verify the HCV status and contact details of the individual and wrote to each individual’s GPs to inform them that they would be contacting their patients to offer confirmatory testing (HCV RNA) and assessment for HCV treatment, unless the GP raised concerns. The Memorandum of Understanding (MoU) stipulated that ODNs were responsible for local information and clinical governance, including provision of appropriate care pathways.

### Evaluation

An evaluation of the re-engagement exercise was developed with four key phases: phase 1: baseline survey (through structured interviews) of the capacity of ODNs and their plans to use the patient lists for case-finding initiatives [17]; phase 2: quantitative assessment with process and outcome indicators; phase 3: qualitative assessment of public and professional perspectives of the re-engagement exercise; and phase 4: economic assessment of the cost effectiveness of the intervention.

This paper focuses on phase 2. Process indicators included whether contact was made with an individual, and the reasons why contact was not made. Outcome indicators include treatment uptake, and reasons for non-treatment (e.g., PCR negative, previous treatment, or death). (Table 1) To measure process and outcome indicators, ODNs were asked to (i) complete standardised monitoring and evaluation spreadsheets (Supplementary information 1), and (ii) to flag ‘re-engagement exercise’ as the reason for referral on the NHSE HCV patient registry and treatment outcome database.

**Table 1:** Definition of re-engagement process and outcomes indicators

Process	Definition
Contacted	An individual was considered to have been contacted if an engagement letter was sent or a phone call was made, and this was indicated in the comments field (in returned spreadsheets). This included where a letter was not delivered e.g., where the letter was returned to sender.
Not contacted	Any of the following: - Where an ODN reported that a person had not been contacted - Where an ODN did not have up-to-date contact details - Where a person had transferred ODN - Where a person was already known to the ODN but not on treatment - Where the ODN indicated they were awaiting a GP response* or where an individual was inappropriate to contact
Outcome	**Definition**
Treated since re-engagement exercise launch	People who have a record in the NHSE treatment registry from 2018 onwards.
Not treated since re-engagement	People with no record in the NHSE treatment registry since 2018. No evidence of treatment since the re-engagement exercise commenced.
Inappropriate to contact	People with complex lifestyles which make treatment challenging, where a decision not to treat had been made by the Multidisciplinary team (MDT), where a person was under palliative care or if a GP objected to contact being made.
Require follow-up	People who responded to ODN contact during the study but are yet to present for testing or have not yet started treatment.
Declining to engage	People who responded to ODN contact during the study and declined testing or treatment and people already known to the ODN prior to the exercise who have yet to commence treatment.
Treated before study	People who have a record in the NHSE treatment registry in 2017 or earlier.
PCR negative	People who were reported as PCR negative by an ODN.
Deceased	If a person was reported as deceased either through ONS mortality data or ODN data.
Awaiting bloodwork	People who had blood drawn by a GP or ODN but for whom results were not yet available when ODNs shared their data.
Transferred	People reported as having moved their treatment to another ODN.
Emigrated	People who were reported to have moved abroad.
Liver related events	People who were reported to have received a liver transplant, who were experiencing end stage liver disease, or who had a Hepatocellular carcinoma (HCC) diagnosis and were still alive.
Unknown status	People who remained unresolved following the re-engagement exercise. This includes people with no contact details, who did not respond to contact from the ODN, who had moved out of the area, or for whom the ODN had no records.

* Awaiting GP response – Where GPs were yet to indicate whether a person was appropriate to contact

The exercise was disrupted by the COVID pandemic with data returned between March and August 2022. Eleven of the 22 ODNs returned the data in monitoring and evaluation spreadsheets and in quarterly reports to NHSE on activity. Because of low response or missing data in the monitoring and evaluation spreadsheets from some ODNs, we supplemented information (for all ODNs including those that provided partial information and those that did not respond) by re-linking individuals on the lists: (i) to the NHSE registry to identify those who had received treatment after the list was shared with ODNs and/or had ‘re-engagement exercise’ flagged as the reason for referral and (ii) to the ONS deaths database to determine any individuals who had subsequently died.

### Data analysis

For individuals included in the final lists to ODNs, we calculated counts and proportions of different socio-demographic characteristics.

We subdivided the dataset into three groups based on individuals with data reported on: (i) both outcomes and processes (e.g., whether contact was attempted); (ii) outcomes but not processes; and (iii) no outcomes or processes. We report on outcomes from the re-engagement exercise for the whole cohort and for these three groups. We also present flow charts and outcomes by these three groups.

For individuals remaining with an unknown outcome, we report on the distribution of unknown status by age, sex, and year of diagnosis.

We also present counts and proportions for ICD-10 causes of death and contributory factors as reported in ONS data. Logistic regression models were used to determine factors associated with receipt of treatment and death. Bivariate analyses were conducted with variables which could have a plausible association. All variables with *p* < 0.1 were included in the multivariable model with parsimony achieved using Wald tests.

### Ethics

Doctors and laboratory directors working in the private or public sectors are mandated by law to report any new diagnoses of HCV as it is a notifiable organism (however it is unknown whether this stipulation is always followed) [18]. The UKHSA collects this information for disease surveillance and to control and prevent the spread of infectious diseases under Sect. 251 of the NHS Act 2006 and the Health Service (Control of Patient Information) Regulations 2002 (regulation 3 / ‘Sect. 251 support’). This allows UKHSA to process personal confidential data without consent. For this exercise, UKHSA sought specific Caldicott approval to share historic laboratory surveillance data with ODNs. The conservative, deterministic linkage process described above was followed to mitigate information governance risks identified during the ethics review process which included (a) accidental or inadvertent disclosure; (b) incidental or inappropriate notification; (c) incorrect diagnoses due to erroneous test coding or poorer performance of older assays; and (d) missed diagnoses due to underreporting and/or incomplete or incorrect information. In its approval, UKHSA’s Caldicott panel indicated that the information governance and confidentiality risks specified within the application were outweighed by the public health benefits in terms of providing treatment to people who may otherwise suffer morbidity and mortality from untreated HCV related liver disease, and by preventing onward transmission of HCV.

Prior to release of patient identifiable data to the ODNs, each ODN signed a MoU with data sharing agreement which outlined that these data should be used solely for the purpose for which special Caldicott permission was received, and not, for example, used for research or shared with academic or commercial entities. The MoU also restated the recipient’s responsibilities about data security, storage and legitimate sharing of data with those involved in direct patient care, as well as the steps that needed to be taken to mitigate information governance risks.

## Results

### Re-engagement lists provided to ODNs

Re-engagement lists varied in size ranging from 1,050 individuals sent to the Leicester ODN to 5,429 individuals sent to the Greater Manchester and Eastern Cheshire ODN (Supplementary information 2).

### Demographic characteristics of patients on lists provided to ODNs

Of 55,329 individuals included in the re-engagement exercise, 36,779 (66.5%) were males, the group had a median age of 51 years (IQR:43, 59) at the time of analysis (2023), 47,668 (86.2%) were diagnosed before 2016, and 11,148 (20.2%) were resident in London. (Table 2)

**Table 2:** Demographic characteristics and outcomes disaggregated by if an ODN returned data

	Total	ODN returned data	No ODN data
	55,329	25,813	29,516
Demographics	*N* (%)	*N* (%)	*N* (%)
Sex			
Female	18,550 (33.5)	8,738 (33.9)	9,812 (33.2)
Male	36,779 (66.5)	17,075 (66.1)	19,704 (66.8)
Age (years)			
< 25	835 (1.5)	346 (1.3)	489 (1.7)
25–34	2,096 (3.8)	960 (3.7)	1,136 (3.9)
35–44	12,638 (22.8)	5,424 (21.0)	7,214 (24.4)
45–54	17,861 (32.3)	8,054 (31.2)	9,807 (33.2)
55–64	14,000 (25.3)	6,888 (26.7)	7,112 (24.1)
65+	7,899 (14.3)	4,141 (16.0)	3,758 (12.7)
Year of diagnosis			
1993–2000	2,926 (5.3)	1,528 (5.9)	1,398 (4.7)
2001–2005	8,230 (14.9)	3,531 (13.7)	4,699 (15.9)
2006–2010	15,531 (28.1)	7,125 (27.6)	8,406 (28.5)
2011–2015	20,981 (37.9)	10,056 (39.0)	10,925 (37.0)
2016–2017	7,656 (13.8)	3,570 (13.8)	4,086 (13.8)
Missing	5 (0.01)	3 (0.01)	2 (0.01)
Region of residence			
North-West	10,796 (19.5)	2,763 (10.7)	8,033 (27.2)
North-East	9,319 (16.8)	3,228 (12.5)	6,091 (20.6)
Midlands & East	11,784 (21.3)	3,593 (13.9)	8,191 (27.8)
London	11,148 (20.2)	5,070 (19.6)	6,078 (20.6)
South-West	4,912 (8.9)	4,912 (19.0)	0 (0)
South-East	7,370 (13.3)	6,247 (24.2)	1,123 (3.8)
Outcomes			
Unknown status	33,349 (60.3)	11,466 (44.4)	21,883 (74.1)
Deceased	2,990 (5.4)	1,367 (5.3)	1,623 (5.5)
Treated since study	7,442 (13.4)	3,381 (13.1)	4,061 (13.8)
PCR negative	6,435 (11.6)	6,435 (24.9)	
Treated before study	4,195 (7.6)	2,246 (8.7)	1,949 (6.6)
Transferred	167 (0.3)	167 (0.6)	
Awaiting bloodwork	35 (0.1)	35 (0.1)	
Require follow-up	276 (0.5)	276 (1.1)	
Declined to engage	411 (0.7)	411 (1.6)	
Decision not to treat	29 (0.1)	29 (0.1)	

### Reporting by ODNs

**Fig. 2:**
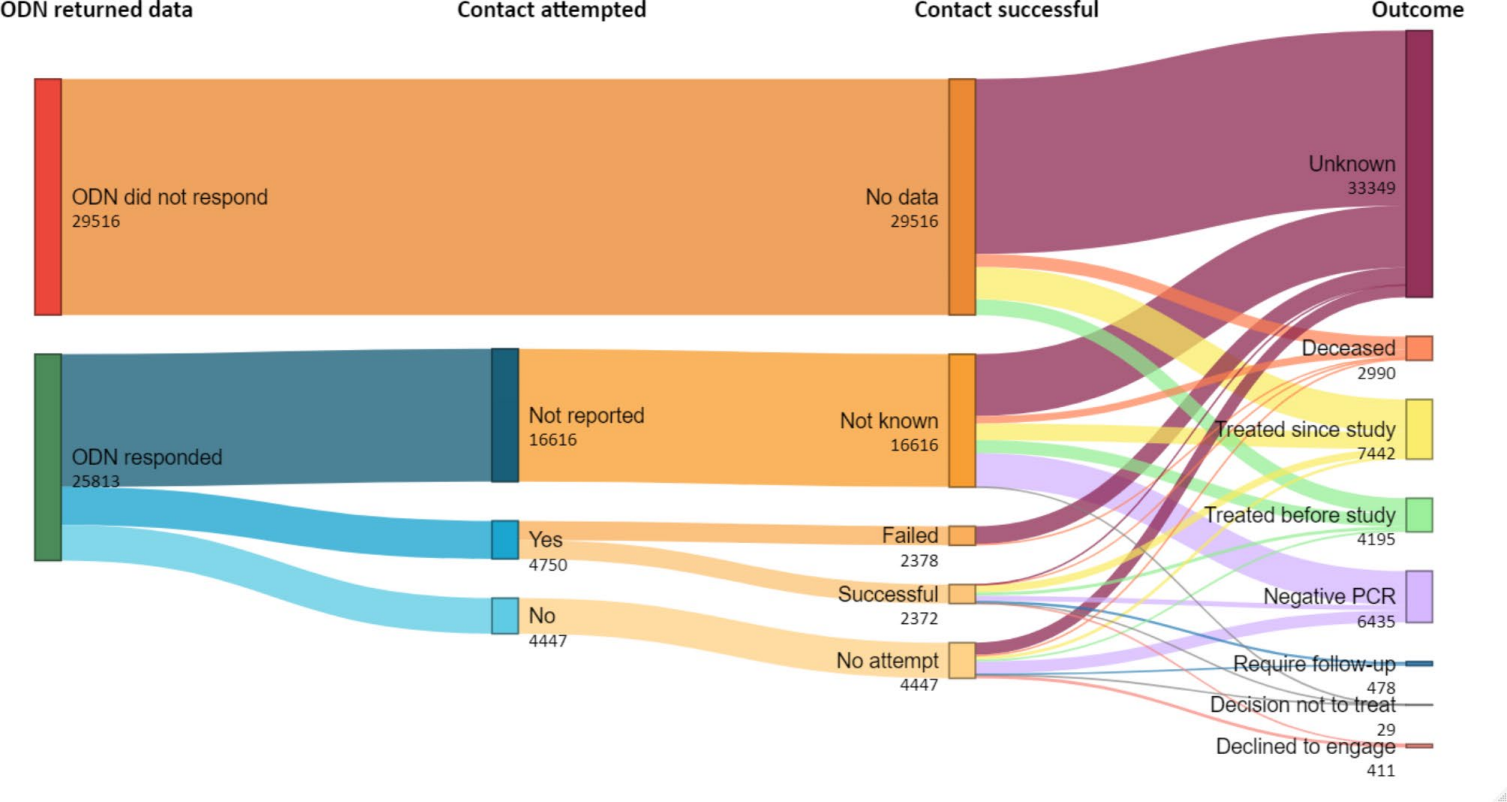
Alluvial plot of the cascade of the re-engagement exercise

Figure 2 summarises the processes and outcomes of the re-engagement exercise. The 11 ODNs that returned data accounted for 25,813 (46.7%) of all the individuals included in the re-engagement exercise. (Table 2) Returned data varied in detail and completeness with two ODNs returning outcome data but no process data (e.g., number of people contacted).

Of eleven ODNs that did not return data, 4 (36.4%) reported preliminary findings in quarterly ODN reports, and 1 (9.1%) had published results from the re-engagement exercise which showed significant variation in its implementation [19]. 

### Process indicators

Of 25,813 individuals from ODNs that returned data, 9,197 (35.6%) had process indicators reported in their record of whom 4,750 (51.6%) were contacted by their ODN. Supplementary Fig. 1 reports on outcomes for individuals with process indicators. Of these, initial investigations excluded 163 individuals who had died and 17 children, 2,317 (48.8%) responded, 2,215 (46.6%) did not respond (115 letters returned to the ODN) and 38 (0.8%) could not be further engaged for multiple reasons. Of the 4,447 with process indicators who had not been contacted, reasons for no contact included: known to be PCR negative (1,612, 36.2%); receipt of treatment before the exercise (264, 5.9%); known to ODN but no evidence of treatment (413, 9.3%); known to ODN and treated (377, 8.5%); transferred care (167, 3.8%); awaiting results (4, < 0.1%); GP indicated inappropriate to contact (12, 0.3%); emigrated (3, < 0.1%). A further 108 (2.4%) individuals had not been contacted as the ODN was still awaiting a GP response. Finally, 1,387 individuals could not be contacted by the ODN either because ODNs did not have up-to-date contact details (513, 11.5%), or they remained unknown to the ODN (no record found) and were therefore considered to be ‘not engaged’ with services (874, 19.6%) (Supplementary Fig. 1).

### Re-engagement outcomes

#### All ODNs

Table 3 summarises outcomes for all individuals. Of 55,329 individuals included in the re-engagement exercise, as of August 2022, 7,442 (13.4%) had accessed treatment since the re-engagement exercise commenced, 2,990 (5.4%) were found to have died, 6,435 (11.6%) were reported as PCR negative (96% of whom had no previous treatment records^1^), 4,195 (7.6%) had prescription data indicating treatment before the exercise commenced or were reported as previously treated by their ODN, 411 (0.7%) declined to engage, 276 (0.5%) had not yet attended a planned ODN appointment, 167 (0.3%) were reported to have transferred their treatment elsewhere, 35 (0.1%) were awaiting blood test results, and for 29 a decision had been made not to treat (12 (< 0.1%) had emigrated, 9 (< 0.1%) were inappropriate to contact and 8 (< 0.1%) had a liver related event). The remaining 33,349 (60.3%) people had an unknown status (547 of these were children). (Table 3)

**Table 3:** Crosstabulation of demographic characteristics and outcomes of individuals included in the re-engagement exercise. (∼ require follow-up refers to individuals who responded to contact from the ODN but had not yet received treatment when data was sent, * decision not to treat includes emigrations and people who were inappropriate to contact)

		Outcomes									
		**Treated**	**Not treated**								
	**Total**	**Treated since study**	**Treated before study**	**Deceased**	**PCR negative**	**Transferred**	**Awaiting bloodwork**	**Require follow-up~**	**Refused to engage**	Decision not to treat*	**Unknown status**
	55,329	7,442 (13.4)	4,195 (7.6)	2,990 (5.4)	6,435 (11.6)	167 (0.3)	35 (0.1)	276 (0.5)	411 (0.7)	29 (0.1)	33,349 (60.3)
Demographics	*N* (%)	*N* (%)	*N* (%)	*N* (%)	*N* (%)	*N* (%)	*N* (%)	*N* (%)	*N* (%)	*N* (%)	*N* (%)
Sex											
Female	18,550 (33.5)	1,947 (26.2)	1,241 (29.6)	886 (29.6)	2,475 (38.5)	59 (35.3)	11 (31.4)	86 (31.2)	145 (35.3)	10 (34.5)	11,690 (35.1)
Male	36,779 (66.5)	5,495 (73.8)	2,954 (70.4)	2,104 (70.4)	3,960 (61.5)	108 (64.7)	24 (68.6)	190 (68.8)	266 (64.7)	19 (65.5)	21,659 (64.9)
Age (years)											
< 25	835 (1.5)	21 (0.3)	25 (0.6)	4 (0.1)	82 (1.3)	1 (0.6)	0 (0.0)	1 (0.4)	7 (1.7)	0 (0.0)	694 (2.1)
25–34	2,096 (3.8)	377 (5.1)	191 (4.6)	27 (0.9)	185 (2.9)	7 (4.2)	0 (0.0)	14 (5.1)	13 (3.2)	1 (3.4)	1,281 (3.8)
35–44	12,638 (22.8)	2,227 (29.9)	1,061 (25.3)	349 (11.7)	1,385 (21.5)	42 (25.1)	5 (14.3)	54 (19.6)	52 (12.7)	3 (10.4)	7,460 (22.4)
45–54	17,861 (32.3)	2,836 (38.1)	1,389 (33.1)	888 (29.7)	2,010 (31.2)	54 (32.3)	6 (17.1)	79 (28.6)	107 (26.0)	6 (20.7)	10,486 (31.4)
55–64	14,000 (25.3)	1,506 (20.2)	1,016 (24.2)	822 (27.5)	1,779 (27.6)	34 (20.4)	10 (28.6)	86 (31.2)	128 (31.1)	5 (17.2)	8,614 (25.8)
65+	7,899 (14.3)	475 (6.4)	513 (12.2)	900 (30.1)	994 (15.4)	29 (17.4)	14 (40.0)	42 (15.2)	104 (25.3)	14 (48.3)	4,814 (14.4)
Year of diagnosis											
1993–2000	2,926 (5.3)	333 (4.5)	164 (3.9)	190 (6.4)	307 (4.8)	16 (9.6)	7 (20.0)	21 (7.6)	91 (22.1)	0 (0.0)	1,797 (5.4)
2001–2005	8,230 (14.9)	1,027 (13.8)	502 (12.0)	509 (17.0)	752 (11.7)	19 (11.4)	2 (5.7)	69 (25.0)	113 (27.5)	1 (3.4)	5,236 (15.7)
2006–2010	15,531 (28.1)	1,990 (26.7)	1,075 (25.6)	789 (26.4)	1,927 (29.9)	59 (35.3)	14 (40.0)	57 (20.7)	117 (28.5)	11 (37.9)	9,492 (28.5)
2011–2015	20,981 (37.9)	2,814 (37.8)	1,752 (41.8)	1,059 (35.4)	2,663 (41.4)	61 (36.5)	7 (20.0)	98 (35.5)	66 (16.1)	14 (48.3)	12,447 (37.3)
2016–2017	7,656 (13.8)	1,278 (17.2)	702 (16.7)	443 (14.8)	785 (12.2)	12 (7.2)	5 (14.3)	31 (11.2)	24 (5.8)	3 (10.4)	4,373 (13.1)
Missing	5 (0.01)	0 (0.0)	0 (0.0)	0 (0.0)	1 (0.01)	0 (0.0)	0 (0.0)	0 (0.0)	0 (0.0)	0 (0.0)	4 (0.01)
Region of residence											
North-West	10,796 (19.5)	1,770 (23.8)	460 (11.0)	659 (22.0)	530 (8.2)	0 (0.0)	1 (2.9)	55 (19.9)	2 (0.5)	1 (3.4)	7,318 (21.9)
North-East	9,319 (16.8)	1,277 (17.2)	764 (18.2)	498 (16.6)	1,449 (22.5)	0 (0.0)	0 (0.0)	0 (0.0)	0 (0.0)	0 (0.0)	5,331 (16.0)
Midlands & East	11,784 (21.3)	1,652 (22.2)	560 (13.4)	609 (20.4)	739 (11.5)	0 (0.0)	34 (97.1)	98 (35.5)	1 (0.2)	0 (0.0)	8,091 (24.3)
London	11,148 (20.2)	1,023 (13.7)	891 (21.2)	526 (17.6)	787 (12.2)	0 (0.0)	0 (0.0)	0 (0.0)	6 (1.5)	0 (0.0)	7,915 (23.7)
South-West	4,912 (8.9)	611 (8.2)	862 (20.5)	283 (9.5)	1,642 (25.5)	0 (0.0)	0 (0.0)	78 (28.3)	385 (93.7)	22 (75.9)	1,029 (3.1)
South-East	7,370 (13.3)	1,109 (14.9)	658 (15.7)	415 (13.9)	1,288 (20.0)	167 (100.0)	0 (0.0)	45 (16.3)	17 (4.1)	6 (20.7)	3,665 (11.0)

#### ODNs that returned data

Among those where contact was attempted and who responded (2,317/4,750), 939 (40.5%) were treated for HCV after the exercise commenced, 649 (28.0%) were already PCR negative, 418 (18.0%) had received treatment prior to the re-engagement exercise, 276 (11.9%) had not yet received treatment and required further follow-up, 34 (1.5%) were awaiting blood test results, and there was 1 (< 1%) decision not to treat. Of those where contact was attempted, there were a further 38 decisions not to treat (21 refused treatment, 9 were not treated due to medical reasons and 8 had emigrated) (Supplementary Fig. 1).

Of 16,616 individuals with outcomes but no process data, 2,146 (12.9%) had been treated since the beginning of the re-engagement exercise (post-2017), 4,437 (26.7%) were PCR negative, 1,692 (10.2%) had previously received treatment (pre-2017), and 7,585 (45.6%) [11 reported as PCR positive but not treated and 7,574 who remained unknown to the ODN] were considered to be ‘not engaged’ with services (Supplementary Fig. 2).

#### ODNs that did not return data

Through linking to ONS death registrations and the HCV patient registry and treatment outcome database (NHSE registry), of the 29,516 individuals where no process or outcome data was returned, 1,623 (5.5%) had died, 1,949 (6.6%) had been previously treated (pre-2017) and 4,061 (13.8%) had been treated since the commencement of the re-engagement exercise (post-2017) (Supplementary Fig. 3).

### Receipt of treatment

Of 55,329 individuals in the exercise, 7,621 (13.8%) had evidence of treatment after the exercise (7,442 were still alive). Females [aOR: 0.66 (95% CI: 0.63–0.70)], and older individuals [e.g., 55-64-year-olds aOR: 0.66 (0.62–0.71) compared to 45-54-year-olds] were less likely to receive treatment. Those diagnosed after 2015 [aOR: 1.35 (1.25–1.45)] and those living outside London [e.g., North-West aOR: 1.99 (1.83–2.17)] were more likely to receive treatment. (Table 4)

**Table 4:** Factors associated with treatment receipt for individuals included in the re-engagement exercise

	Total	Treated since study	OR (95% CI)	*p*-value	aOR (95% CI)	*p*-value
					(*n* = 55,324)	
Total	55,329	7,621				
Sex						
Female	18,550 (33.5)	1,994 (26.2)	0.67 (0.63, 0.70)	< 0.001	0.66 (0.63, 0.70)	< 0.001
Male	36,779 (66.5)	5,627 (73.8)	Reference		Reference	
Age						
< 25	835 (1.5)	21 (0.3)	0.13 (0.09, 0.21)	< 0.001	0.14 (0.09, 0.21)	< 0.001
25–34	2,096 (3.8)	379 (5.0)	1.14 (1.01, 1.28)	0.03	1.13 (1.00, 1.27)	0.055
35–44	12,638 (22.8)	2,254 (29.6)	1.12 (1.05, 1.19)	< 0.001	1.14 (1.07, 1.22)	< 0.001
45–54	17,861 (32.3)	2,897 (38.0)	Reference		Reference	
55–64	14,000 (25.3)	1,566 (20.5)	0.65 (0.61, 0.69)	< 0.001	0.66 (0.62, 0.71)	< 0.001
65+	7,899 (14.3)	504 (6.6)	0.35 (0.32, 0.39)	< 0.001	0.37 (0.34, 0.41)	< 0.001
Year of diagnosis						
1993–2000	2,926 (5.3)	346 (4.5)	0.84 (0.75, 0.95)	0.005	0.90 (0.80, 1.02)	0.098
2001–2005	8,230 (14.9)	1,058 (13.9)	0.93 (0.86, 1.00)	0.054	0.89 (0.82, 0.96)	0.003
2006–2010	15,531 (28.1)	2,035 (26.7)	0.95 (0.89, 1.01)	0.091	0.90 (0.85, 0.96)	0.002
2011–2015	20,981 (37.9)	2,877 (37.8)	Reference		Reference	
2016–2017	7,656 (13.8)	1,305 (17.1)	1.29 (1.20, 1.39)	< 0.001	1.35 (1.25, 1.45)	< 0.001
Missing	5 (0.01)	0 (0.0)				
Region of residence						
North-West	10,796 (19.5)	1,821 (23.9)	1.95 (1.80, 2.11)	< 0.001	1.99 (1.83, 2.17)	< 0.001
North-East	9,319 (16.8)	1,302 (17.1)	1.56 (1.43, 1.70)	< 0.001	1.46 (1.34, 1.60)	< 0.001
Midlands & East	11,784 (21.3)	1,682 (22.1)	1.60 (1.47, 1.73)	< 0.001	1.54 (1.42, 1.68)	< 0.001
London	11,148 (20.2)	1,052 (13.8)	Reference		Reference	
South-West	4,912 (8.9)	626 (8.2)	1.40 (1.26, 1.56)	< 0.001	1.45 (1.30, 1.61)	< 0.001
South-East	7,370 (13.3)	1,138 (14.9)	1.75 (1.60, 1.92)	< 0.001	1.83 (1.67, 2.01)	< 0.001

### Unknown outcomes

#### All ODNs

Of 55,329 individuals included in the re-engagement exercise, 33,349 (60.3%) continued to have an unknown outcome (547, 1.6% of whom were children). They had a median age of 51 years (IQR: 43, 60), 21,659 (64.9%) were male, and 26,312 (78.9%) were diagnosed between 2006 and 2017, the largest proportion of whom (12,447, 37.3%) were diagnosed between 2011 and 2015.

#### Only ODNs that returned data

For ODNs that returned data, of 25,813 individuals included in the exercise, 11,466 (44.4%) continued to have an unknown status (168 were children). They had a median age of 52 years (IQR: 44, 61), 7523 (65.6%) were male, and 9,117 (79.5%) were diagnosed between 2006 and 2015, the largest proportion (4,396, 38.3%) between 2011 and 2015.

### Mortality

Of 2,990 individuals who had died, 2,104 (70.4%) were males and median age at death was 54 years (IQR: 46, 63). The underlying cause was missing for 515 (17.2%) deaths. The leading single underlying cause of death, where available, was HCC accounting for 183 (7.4%) of deaths with a reported underlying cause, liver disease accounted for 300 (12.1%), and viral hepatitis for 88 (3.6%) of deaths. There were 571 (19.1%) reported liver related deaths, with HCV indicated as a contributory cause for 271/571 (47.5%). HCV was a contributory factor for 457 (15.3%) of all deaths while HCC and ESLD were contributory factors for 222 (7.4%) and 227 (7.6%) of deaths respectively (Supplementary information 3). In logistic regression models, we found that older individuals, males, those diagnosed after 2015, and those living outside London were more likely to have died (Supplementary information 4)

## Discussion

We report on a nation-wide exercise utilising national diagnostic testing surveillance data and established clinical networks to re-engage individuals who previously tested positive for HCV and to offer treatment to those confirmed HCV RNA positive. Following this collaborative effort between UKHSA and the NHSE ODNs, 7,442 (13% increasing to 18% when we exclude previously treated, PCR negative and those who died) individuals who were previously not engaged in care were prescribed HCV treatment. These individuals are estimated to represent 10% of the total number of individuals treated in England since 2015. We also found that 2,990 (5%) individuals had died of whom 15% had HCC and/or ESLD recorded as a contributing factor on their death certificate. Overall the exercise was unable to directly re-engage 33,349 (60%) of identifiable individuals with known HCV antibody or RNA positivity thought to be alive.

Our overall findings are similar to those published by Birmingham ODN which reported modest (11.3%) response rates to letters and low (25%) confirmed SVR numbers [19]. Similar exercises conducted in Wales [20], Netherlands [21, 22], and France [23] reported re-engagement rates of 23% and treatments rates ranging from 8 to 15%. The Relink program used medical record review to identify eligible participants, re-engaged 33% of 11,163 participants in six countries, and treated 6% [24, 25]. Similar to this exercise, the Trap Hep C programme in Iceland addressing an infected population of 1,100 compared to 81,000 in England [4], used cross-referenced surveillance and laboratory data and managed to re-engage all 24 participants achieving SVR12 for 83% [26]. A clinical trial in the Canary Islands found that phone calls were more effective than letters for re-engaging people previously diagnosed with HCV [27]. Participants were less likely to re-engage if they had a history of drug use, tested in the pre-DAA era, and had no prior specialist evaluation [28]. Other studies have used a range of approaches to encourage re-engagement in HIV care, including text messaging and physical tracing. [29–33]

Individuals treated since the exercise included individuals already known to the ODN, and individuals not known, who might have been unaware of their infection, aware of their infection but not engaged with healthcare services, and/or unaware of the emergence of new, better tolerated and more effective treatments. Our findings suggest that Londoners, females, the very young and very old might benefit from a targeted effort to get them onto treatment. The number of individuals treated suggests that using national surveillance data as the basis for patient re-engagement exercises has some utility, but requires further interrogation of additional data sources to refine and validate the data, engagement from all stakeholders, with extensive follow up and local data checks by the ODNs. In some instances, despite positive re-engagement some individuals refused treatment. It is important that these individuals have continuing support and access to treatment should they change their mind.

Approximately 12% of individuals included in the exercise were found to be PCR negative of whom only 4% had evidence of treatment. This could reflect several mechanisms including spontaneous clearance of infection, [34] treatment outside the NHS (privately or outside England), or failure to record treatment. Given the mix of antibody and PCR tests in the laboratory surveillance dataset, the study would have included some individuals without an active infection. For example, a study conducted in GP practices in Southwest England found only 40% of participants recorded as antibody positive were confirmed to be vireamic [35]. This group might have been less likely to engage with the exercise if they knew they had cleared HCV. The introduction of routine reflex PCR testing of antibody positive samples and point-of-care PCR testing in England [19, 36] should eliminate this as an issue for future similar exercises.

The exercise also revealed that 5.4% of individuals included in the exercise had died. A substantial proportion had HCV, ESLD and/or HCC as either a direct or contributory cause of death. Liver related deaths with HCV reported as a contributory cause were similar to those reported in another study [37]. Similar to other studies, we found that males, and older individuals were more likely to have died [38, 39]. Those outside London were also more likely to have died consistent with mortality trends from ONS [40], as were those diagnosed more recently. Those diagnosed recently could have a higher proportion of active injection drug use which is the primary route of infection in England [6]. However, we did not have access to this or other data such as biomarkers for disease progression, social economic status, and other health risk behaviours (e.g. alcohol use) which are consistent predictors of mortality in people with HCV [41, 42]. The advent of DAAs has rendered HCV a curable disease in the vast majority of cases, so these deaths might have been avoided with earlier engagement in care. These findings illustrate the importance of the test and treat models, and ongoing work by NHSE to simplify the care pathway to ensure that people testing positive for HCV RNA have quick and easy access to treatment.

We found that 60% of those on the lists shared with ODNs still had an unknown outcome. The majority of this group were males, 40 years or older, most of whom had been diagnosed between 2011 and 2015. However, females contributed a larger proportion: 63.0% of females vs. 58.9% of males did not re-engage. Another study reported lower re-engagement for individuals diagnosed in the era preceding widespread use of DAAs [28]. In our study, varied levels of re-engagement likely represent implementation and individual-level challenges. Implementation challenges include varying ODN engagement with 11 of the 22 ODNs not reporting data, thus limiting our ability to fully evaluate the exercise. Secondly, there was significant heterogeneity in the implementation approaches used by ODNs. In the phase 1 evaluation, ODNs reported several obstacles including a lack of dedicated human resources and funding which may have contributed to this variability. Thirdly, due to varying data completeness, many individuals could not be found in any of the records ODNs cross-checked or could not be reached due to a lack of up-to-date contact details. Studies suggest that better infrastructure could improve re-engagement exercises, including simple fixes such as regular data sharing between health facilities [43]. Many printed letters, which were the main mode of contacting individuals in the exercise, were unanswered or not delivered, and letters have been shown to be less effective than other methods e.g., phone calls [27]. Finally, the exercise was designed to make successful re-engagement independent of GP involvement as GPs are often overburdened and have competing priorities [44, 45]. However, the largest proportion of diagnoses in laboratory surveillance are made through primary care [46], and studies have shown higher treatment initiation and SVR rates for participants treated in primary care [47]. Closer integration of primary care could result in better outcomes as some studies in primary care have shown moderate success [35]. Additionally, there are several initiatives being implemented to reach this population including opt-out bloodborne virus testing in emergency departments [48, 49], and targeted testing in GPs [35, 50]. 

Individual barriers affecting re-engagement could include anticipated stigma [51], mistrust of institutions charged with their care [52], and the mobility and transience of some people affected by HCV as demonstrated in other studies [53, 54]. 

Factors such as dissatisfaction with services, insufficient knowledge of HCV and treatment outcomes, complex needs, competing priorities and concerns about treatment side effects may both result in disengagement and affect re-engagement [55]. Service design should minimise barriers and maximise engagement opportunities, using approaches informed by behavioural science. Services must adapt to cater to transient and underserved populations and more complex cases using a more patient-centred approach [56, 57]. Awareness campaigns are also necessary to educate the wider public about new treatments to enable them to objectively assess their own risk [58]. 

A qualitative evaluation of the re-engagement process might help identify factors leading to more effective engagement of ODNs such as monetary incentivisation, [50] and more effective networked data infrastructure. Implementation could be improved with more quality control and refining of datasets before they are shared, support for ODNs to perform data cross-checks, capacity building, and implementation toolkits to facilitate future exercises.

Strict criteria were used to minimise data errors in creating the lists provided to the ODNs and mitigate information governance risks. A further 69,472 individuals were excluded from the initial exercise due to lack of sufficient identifiers or data inconsistencies (Fig. 1) highlighting the importance of comprehensive data requiring further investment.A proportion of these individuals are likely to be viraemic and, because they are so numerous, without treatment, the goal of HCV elimination will remain challenging [4]. 

Reasons for the variation in ODN response are not well understood and merit further investigation. Qualitative in-depth interviews are planned to understand the causes of variation to gain insights that could optimise future re-engagement efforts. There is a pressing need to involve patients to understand their experiences of the exercise. While ODNs indicated that the exercise appeared acceptable to patients and reported no adverse consequences, [17] qualitative research is planned to explore participants’ experiences. A separate exercise to re-engage children is also being conducted by the paediatrics team.

There are several key strengths of the re-engagement exercise. Firstly, the data used was diagnostic testing data for England which was made notifiable for HCV in 2010 and therefore should include all diagnostic tests for HCV from that time, as well as a majority of those reported prior to 2010. Secondly, multiple healthcare databases were used to build the re-engagement lists, allowing for triangulation of data especially concerning HCV diagnoses, treatment and death, further enhanced by local checks undertaken by ODNs. Data linkage permitted the creation of a more comprehensive database than would have been obtained relying solely on ODN reports [59, 60]. 

However, the exercise also had some limitations. Firstly, there was varying engagement from ODNs. As such, our analyses were restricted by the amount and quality of data returned by ODNs. Secondly, information governance issues especially between laboratories and ODNs significantly hampered the exercise. Third, many individuals included on the basis of a positive HCV antibody test may have cleared HCV infection spontaneously or through private treatment. Fourth, we cannot attribute all treatment initiations reported to the re-engagement exercise as this reason was not consistently recorded in the NHSE registry. Finally, it is important to acknowledge the impact of COVID pandemic on the exercise.

In conclusion, this exercise was a substantial and extensive undertaking facilitated by access to key data resources and the participation of multiple organisations. The use of HCV surveillance data to re-engage individuals into care resulted in a sizeable number of people with known HCV infection accessing treatment. Further work is needed to investigate how those engaged differ from those whose infection and treatment status remain unknown. Repeat re-engagement exercises with improved implementation and alternative, complementary elimination strategies should be considered.

## Electronic supplementary material

Below is the link to the electronic supplementary material.

Supplementary Material 1

## Data Availability

No datasets were generated or analysed during the current study.

## Footnotes

1 Only 258 (4%) of individuals reported as PCR negative had evidence of treatment in the NHSE registry [19 (7.3%) 2017 or earlier, 156 (60.5%) in 2018 and 83 (32.2%) missing].
