# Supplementary material for: Results from a retrospective case finding and re-engagement exercise for people previously diagnosed with hepatitis C virus to increase uptake of directly acting antiviral treatment
Source: BMC Public Health. 2024 Sep 6;24:2427. doi: 10.1186/s12889-024-19919-3 (PMC11378625; doi:10.1186/s12889-024-19919-3)

**Supplementary information:**

**Supplementary information 1: Standardised data requested from ODNs to evaluate the re-engagement exercise.**

| Field | Options |
| --- | --- |
| PCR status of patient according to local data checks | - Positive - Negative - Unknown |
| Source of local data checks | - Lab system - GP - Hospital records - Clinical database - Other (specify) |
| If PCR negative, reason | - Treated - Spontaneous clearance - Unknown |
| Who is making initial contact with the patient? | - GP - ODN - Other (specify) |
| Was contact with patient attempted? | - No, GP advised no contact. - No, insufficient details to contact patient. - No, but attempted contact is planned. - Yes, contact attempted. - Other (specify) |
| Outcome of attempted contact with patient | - Patient responded, agreed to confirmatory test. - Patient responded, declined further involvement. - Patient did not respond. - Patient responded, but subsequently lost to follow-up. - Other (specify) |
| PCR result following patient contact | - Positive - Negative - Unknown |
| Additional comments/notes | e.g., patient not anti-HCV positive, patient not found, number of attempts to contact patient |

**Supplementary information 2: Number of individuals on re-engagement lists by ODN.**

| Operational Delivery Network (ODN) | Region | Re-engagement list, n (%) |
| --- | --- | --- |
| Greater Manchester and Eastern Cheshire | North | 5,429 (9.8) |
| Birmingham | Midlands and East | 4,862 (8.8) |
| Eastern Hep. Network | Midlands and East | 3,593 (6.5) |
| South Thames Hep. Network | London | 3,492 (6.3) |
| West Yorkshire | North | 3,228 (5.8) |
| Bristol And Severn | South | 3,135 (5.7) |
| West London | London | 2,865 (5.2) |
| Cheshire And Merseyside | North | 2,763 (5.0) |
| Lancashire And South Cumbria | North | 2,604 (4.7) |
| North Central London | London | 2,586 (4.7) |
| South Yorkshire | North | 2,338 (4.2) |
| Nottingham | Midlands and East | 2,279 (4.1) |
| Barts | London | 2,205 (4.0) |
| Wessex Hep C ODN | South | 2,159 (3.9) |
| Northeast And Cumbria | North | 2,123 (3.8) |
| Southwest Peninsula | South | 1,777 (3.2) |
| Sussex Hep. Network | South | 1,701 (3.1) |
| Humberside And North Yorkshire | North | 1,630 (2.9) |
| Thames Valley Hep C Network | South | 1,225 (2.2) |
| Surrey Hep. Services | South | 1,162 (2.1) |
| Kent Network Via Kings | South | 1,123 (2.0) |
| Leicester | Midlands and East | 1,050 (1.9) |
| Total |  | **55,329** |

**Supplementary information 3:**  Causes of death.

| Underlying cause of death | All  N (%) | HCV contributory  n (%) | HCC contributory  n (%) | ESLD contributory  n (%) |
| --- | --- | --- | --- | --- |
| HCC | 183 (6.1) | 95 (20.8) | 183 (82.4) | 16 (7.0) |
| ESLD | 57 (1.9) | 11 (2.4) | 1 (0.5) | 57 (25.1) |
| Viral hepatitis | 88 (2.9) | 88 (19.3) | 3 (1.4) | 37 (16.3) |
| Non-alcoholic Liver Disease | 97 (3.2) | 23 (5.0) | 7 (3.1) | 24 (10.6) |
| Alcoholic Liver Disease | 146 (4.9) | 54 (11.8) | 7 (3.1) | 57 (25.1) |
| External causes* | 423 (14.2) | 15 (3.3) | 0 (0) | 4 (1.8) |
| Other cancers | 349 (11.7) | 27 (5.9) | 3 (1.4) | 3 (1.3) |
| Other** | 1,132 (37.9) | 123 (26.9) | 12 (5.4) | 24 (10.6) |
| Missing | 515 (17.2) | 21 (4.6) | 6 (2.7) | 5 (2.2) |
| Total | 2,990 | 457 | 222 | 227 |

* External causes includes suicide, accidental poisoning and overdoses, homicide, transport accidents.

** Other (411 circulatory (13.7), 308 (10.3) respiratory, 101 (3.4) mental behavioural, 74 (2.5) digestive)

**Supplementary information 4:** Factors associated with mortality for individuals included in the re-engagement exercise

|  | All | Deceased | OR (95% CI) | p-value | aOR (95% CI) | p-value |
| --- | --- | --- | --- | --- | --- | --- |
|  |  |  |  |  | (n=55,324) |  |
| Total | 55,329 | 2,990 |  |  |  |  |
| Sex |  |  |  |  |  |  |
| Female | 18,550 (33.5) | 886 (29.6) | 0.83 (0.76, 0.90) | <0.001 | 0.84 (0.78, 0.91) | <0.001 |
| Male | 36,779 (66.5) | 2,104 (70.4) | Reference | ___ | Reference | ___ |
| Age |  |  |  |  |  |  |
| <25 | 835 (1.5) | 4 (0.1) | 0.09 (0.03, 0.25) | <0.001 | 0.09 (0.03, 0.24) | <0.001 |
| 25-34 | 2,096 (3.8) | 27 (0.9) | 0.25 (0.17, 0.37) | <0.001 | 0.24 (0.16, 0.36) | <0.001 |
| 35-44 | 12,638 (22.8) | 349 (11.7) | 0.54 (0.48, 0.62) | <0.001 | 0.54 (0.48, 0.61) | <0.001 |
| 45-54 | 17,861 (32.3) | 888 (29.7) | Reference | __ | Reference | ___ |
| 55-64 | 14,000 (25.3) | 822 (27.5) | 1.19 (1.08, 1.31) | <0.001 | 1.23 (1.12, 1.36) | <0.001 |
| 65+ | 7,899 (14.3) | 900 (30.1) | 2.46 (2.23, 2.71) | <0.001 | 2.63 (2.38, 2.90) | <0.001 |
| Year of diagnosis |  |  |  |  |  |  |
| 1993-2000 | 2,926 (5.3) | 190 (6.4) | 1.31 (1.11, 1.53) | 0.001 | 0.95 (0.80, 1.11) | 0.511 |
| 2001-2005 | 8,230 (14.9) | 509 (17.0) | 1.24 (1.11, 1.38) | <0.001 | 1.02 (0.91, 1.14) | 0.779 |
| 2006-2010 | 15,531 (28.1) | 789 (26.4) | 1.01 (0.92, 1.11) | 0.888 | 0.88 (0.80, 0.97) | 0.012 |
| 2011-2015 | 20,981 (37.9) | 1,059 (35.4) | Reference | __ | Reference | ___ |
| 2016-2017 | 7,656 (13.8) | 443 (14.8) | 1.16 (1.03, 1.29) | 0.013 | 1.27 (1.13, 1.42) | <0.001 |
| Missing | 5 (0.01) | 0 (0.0) | __ | __ | __ | __ |
| Region of residence |  |  |  |  |  |  |
| North-West | 10,796 (19.5) | 659 (22.0) | 1.31 (1.17, 1.48) | <0.001 | 1.55 (1.37, 1.75) | <0.001 |
| North-East | 9,319 (16.8) | 498 (16.7) | 1.14 (1.00, 1.29) | 0.041 | 1.56 (1.37, 1.78) | <0.001 |
| Midlands & East | 11,784 (21.3) | 609 (20.4) | 1.10 (0.98, 1.24) | 0.117 | 1.31 (1.16, 1.48) | <0.001 |
| London | 11,148 (20.2) | 526 (17.6) | Reference | __ | Reference | ___ |
| South-West | 4,912 (8.9) | 283 (9.5) | 1.23 (1.06, 1.43) | 0.005 | 1.38 (1.19, 1.61) | <0.001 |
| South-East | 7,370 (13.3) | 415 (13.9) | 1.20 (1.06, 1.37) | 0.006 | 1.29 (1.12, 1.47) | <0.001 |

**Supplementary Figure 1:** Flow diagram of the re-engagement exercise for individuals with process and outcome data


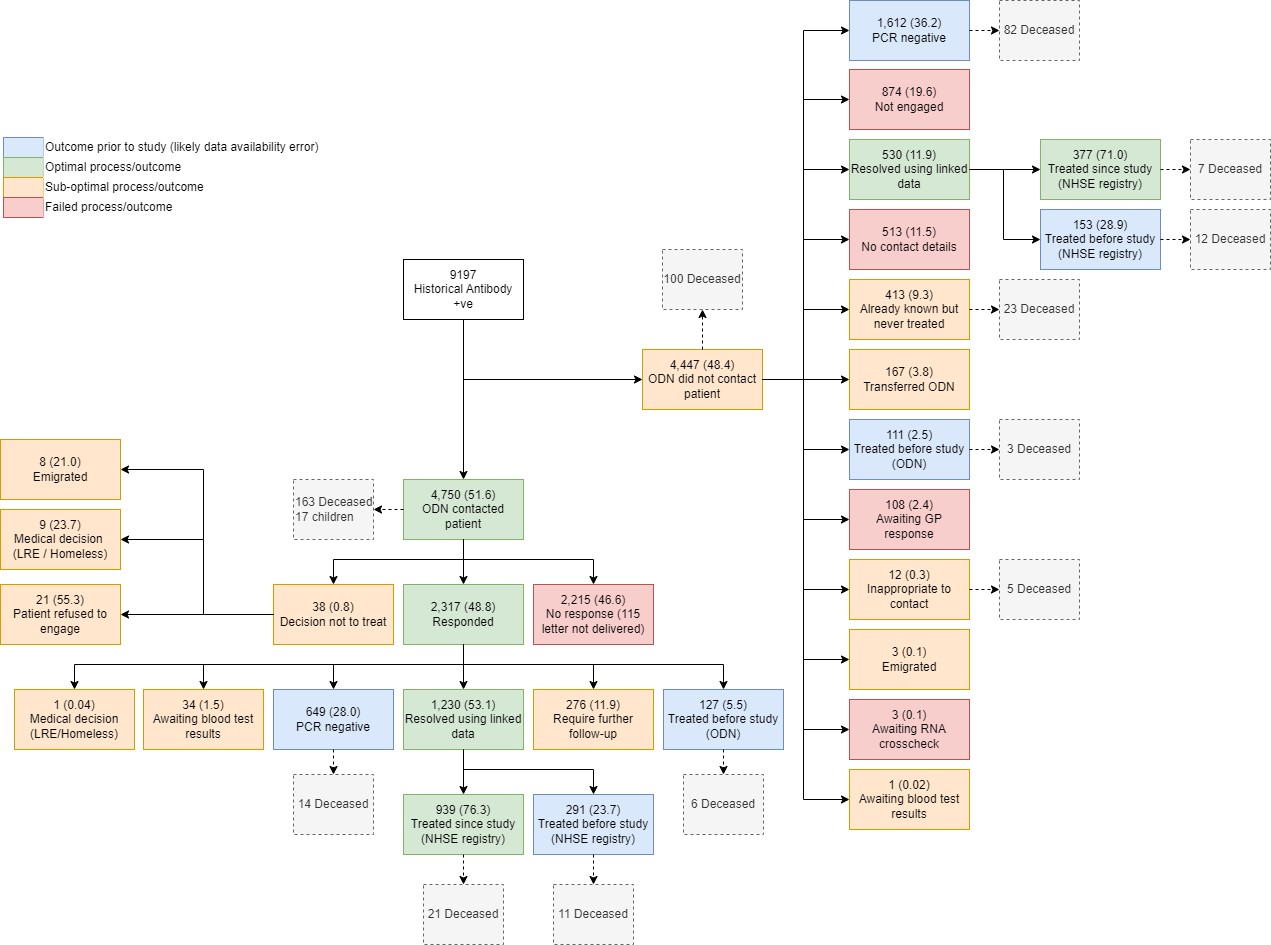


**Supplementary Figure 2:** Flow diagram of the re-engagement exercise for individuals with outcome but not process data.


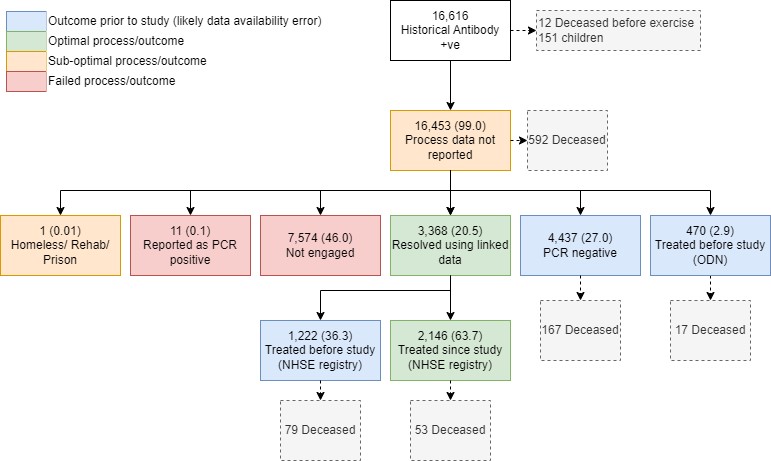


**Supplementary Figure 3:** Flow diagram of the re-engagement exercise for ODNs that did not return either outcome or process data


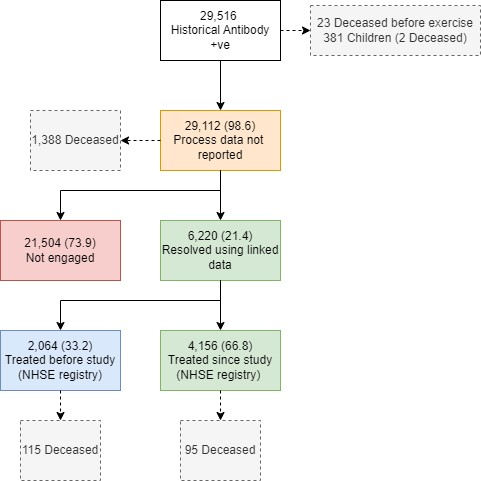

Supplement: Supplementary file 1 — Supplementary Material 1 [file 12889_2024_19919_MOESM1_ESM.docx]
